# Laughing gas-induced psychotic disorder : Case report from Morocco.

**DOI:** 10.1192/j.eurpsy.2023.1366

**Published:** 2023-07-19

**Authors:** B. Zineb, T. Aicha, K. Imane, S. Maria, E. O. Fatima

**Affiliations:** Ar-razi Psychiatric hospital, Faculty of Medecine and Pharmacy, Rabat, Morocco

## Abstract

**Introduction:**

In the current psychopharmacological panorama, the variety of substances capable of inducing an acute psychotic episode and which have entered the habits of drug addicts has rapidly increased. Here we will take the example of nitrous oxide, which in addition to its medical use as a volatile anesthetic, has many applications in the food and automotive industries.Nitrous oxide is today the 7th most popular drug in the world for its euphoric effects.

**Objectives:**

The objective of our work is therefore to present through a case report, where the psychiatric symptoms are important, an overview of the psychiatric effects of the recreational use of nitrous oxide, to sensitize the clinicians, and to finally discuss the implications for psychiatric practice in terms of prevention and screening.

**Methods:**

Case report: To better discuss this infrequent disorder, we will report here the case of a 25-year-old French tourist, with no particular psychiatric or medical history, who was brought back to our training emergency room by the authorities for treatment of psychomotor instability. , verbalization of delusional remarks and insomnia evolving for approximately 02 days, following an excessive and isolated use of nitrogen peroxide bombs, in a festive setting in Marrakech.

The psychiatric interview objectified a dissociative syndrome, a delusional syndrome of persecution and mystical-religious, a hallucinatory syndrome, with impaired judgment and insight. A complete biological assessment, a cerebral TDM as well as a search for drugs in the urine were requested, returned without particularities.

The patient was put on antipsychotics and anxiolytics, with very good clinical evolution and complete resolution of symptoms after 02 days.

**Results:**

Nitrous oxide (N2O; laughing gas) is used clinically as a safe anesthetic (dentistry, ambulance, childbirth) and is appreciated for its anti-anxiety effect. Over the past five years, its recreational use has rapidly increased, particularly in the world of dance and festivals.

Side effects of N2O include transient dizziness, dissociation, disorientation, loss of balance, impaired memory and cognition, and weakness in the legs. In cases of poisoning, accidents such as tripping and falling can occur.

Some fatalities have been reported due to asphyxia (hypoxia). Heavy or sustained use of N2O inactivates vitamin B12, resulting in functional vitamin B12 deficiency and initially causes finger numbness, which can later progress to peripheral neuropathy and megaloblastic anemia. The use of N2O does not appear to be addictive.

**Conclusions:**

Given the generally modest use of N2O and its relative safety, there is no need for legal action. However, (potential) users should be informed of the risk of neurological and hematological effects related to vitamin B12 deficiency in case of intensive use.

**Disclosure of Interest:**

None Declared

